# Calorie intake rather than food quantity consumed is the key factor for the anti-aging effect of calorie restriction

**DOI:** 10.18632/aging.203493

**Published:** 2021-09-07

**Authors:** Yaru Liang, Yuqi Gao, Rui Hua, Maoyang Lu, Huiling Chen, Zhuoran Wang, Liyuan Li, Kaiqiang Hu, Yuemiao Yin, Kang Xu, Hongqi Gao, Qingfei Liu, Ying Qiu, Zhao Wang

**Affiliations:** 1 MOE Key Laboratory of Protein Sciences, School of Pharmaceutical Sciences, Tsinghua University, Beijing 100084, P.R. China; 2 Department of Anesthesiology, School of Medicine, Duke University, Durham, NC 27708, USA; 3 School of Medicine, Tsinghua University, Beijing 100084, P.R. China

**Keywords:** calorie restriction, food quantity, calorie intake, healthspan, fatty acid biosynthesis

## Abstract

Although calorie restriction has been reported to extend lifespan in several organisms, animals subjected to calorie restriction consume not only fewer calories but also smaller quantities of food. Whether it is the overall restriction of calories or the coincidental reduction in the quantity of food consumed that mediates the anti-aging effects is unclear. Here, we subjected mice to five dietary interventions. We showed that both calorie and quantity restriction could improve early survival, but no maximum lifespan extension was observed in the mice fed isocaloric diet in which food quantity was reduced. Mice fed isoquant diet with fewer calories showed maximum lifespan extension and improved health among all the groups, suggesting that calorie intake rather than food quantity consumed is the key factor for the anti-aging effect of calorie restriction. Midlife liver gene expression correlations with lifespan revealed that calorie restriction raised fatty acid biosynthesis and metabolism and biosynthesis of amino acids but inhibited carbon metabolism, indicating different effects on fatty acid metabolism and carbohydrate metabolism. Our data illustrate the effects of calories and food quantity on the lifespan extension by calorie restriction and their potential mechanisms, which will provide guidance on the application of calorie restriction to humans.

## INTRODUCTION

Calorie restriction (CR), commonly defined as a 20-40% reduction in calorie intake, is one of the most effective interventions for the modulation of aging. It is reported that calorie restriction extends both the maximum and average lifespan of many organisms investigated [1, 2], including yeast [3, 4], nematodes [5], fruit flies [6], and mice [7]. In contrast, a high-calorie diet shortens the lifespan of mice and leads to cardiovascular diseases, obesity, and other metabolic disorders associated with aging [8–10]. Daily calorie intake per se is identified as a determinant of longevity, and the source of calories (carbohydrates, fat, or protein) is considered irrelevant [11]. Evidence for this view comes from the following experiments in rats: (1) reducing caloric intake without limiting protein intake prolongs lifespan in rats [12]; (2) Rats fed an isocaloric diet with a reduced mineral content or fat did not show extended lifespan [13]. Nevertheless, in other studies, rats fed isocaloric diets with changed nutritional composition [13–15] or reduced protein [16] showed an extended lifespan. Furthermore, solely reducing the amount of methionine prolongs the lifespan of mice and rats [17]. Thus, reduced calorie intake may not be the key determinant of extended lifespan in rodents by dietary restriction. It was reported that time-limited feeding can prevent metabolic disorders in mice fed a high-fat diet without reducing calorie intake [18]. Apart from the nutritional composition and feeding time, there is also a parameter that we have overlooked in previous studies. Animals subjected to a 20-40% calorie reduction consume not only fewer calories but also a smaller quantity of food. Whether it is the overall restriction of calories or the coincidental reduction in the quantity of food consumed that mediates the anti-aging effects of calorie restriction is unclear. Here, to test whether a reduction in the quantity of food alone or calorie restriction alone can extend a lifespan, we subjected mice to five diet and energy regimens (n=30 per group) wherein the NF group was the normal control group, the LF group solely restricted calorie intake, the HDR group solely restricted quantity intake and the NDR group is the traditionally reported calorie restriction intervention with both calorie and quantity intake restriction. The order of lifespan of mice feeding different regimens are as follows: 125% high-calorie diet fed ad libitum (HF), normal-calorie diet ad libitum (NF), normal-calorie diet at 80% of the normal food quantity (NDR), 125% high-calorie diet at 80% of the normal food quantity (HDR), and 80%-calorie diet fed at the normal food quantity (LF). Apart from lifespan, calorie restriction delays the development of some aging-related diseases including diabetes, cancer, atherosclerosis, neurodegenerative and respiratory failures, thus increasing the healthspan [19, 20], so we also investigated the effect of sole calorie or quantity intake restriction on the healthspan.

Four pathways have been implicated in the mechanism of the calorie restriction effects, such as the insulin-like growth factor (IGF-1)/insulin signaling pathway [21], the adenosine monophosphate (AMP) activated protein kinase pathway [22], sirtuin pathway [23], and the target of rapamycin (TOR) pathway [24]. In this context, we aimed to determine whether food quantity restriction and calorie restriction could give rise to different life extensions according to different pathways. All of the intervention trials were conducted in parallel in the same laboratory, which makes it possible for us to conduct integrated data analysis without systematic variations. Besides, it was reported that middle-aged liver gene expression levels are related to the lifespan under different dietary interventions [25]; therefore, we analyzed the changes in gene expression levels under different dietary interventions and tried to search for common target genes that contribute to lifespan extension. Our results suggest that the enhanced fatty acid biosynthesis and metabolism and inhibited carbon metabolism are key determinants of the lifespan extension after different dietary interventions.

## RESULTS

### Metabolic phenotypes and longevity in mice with different dietary interventions

**Figure 1:**
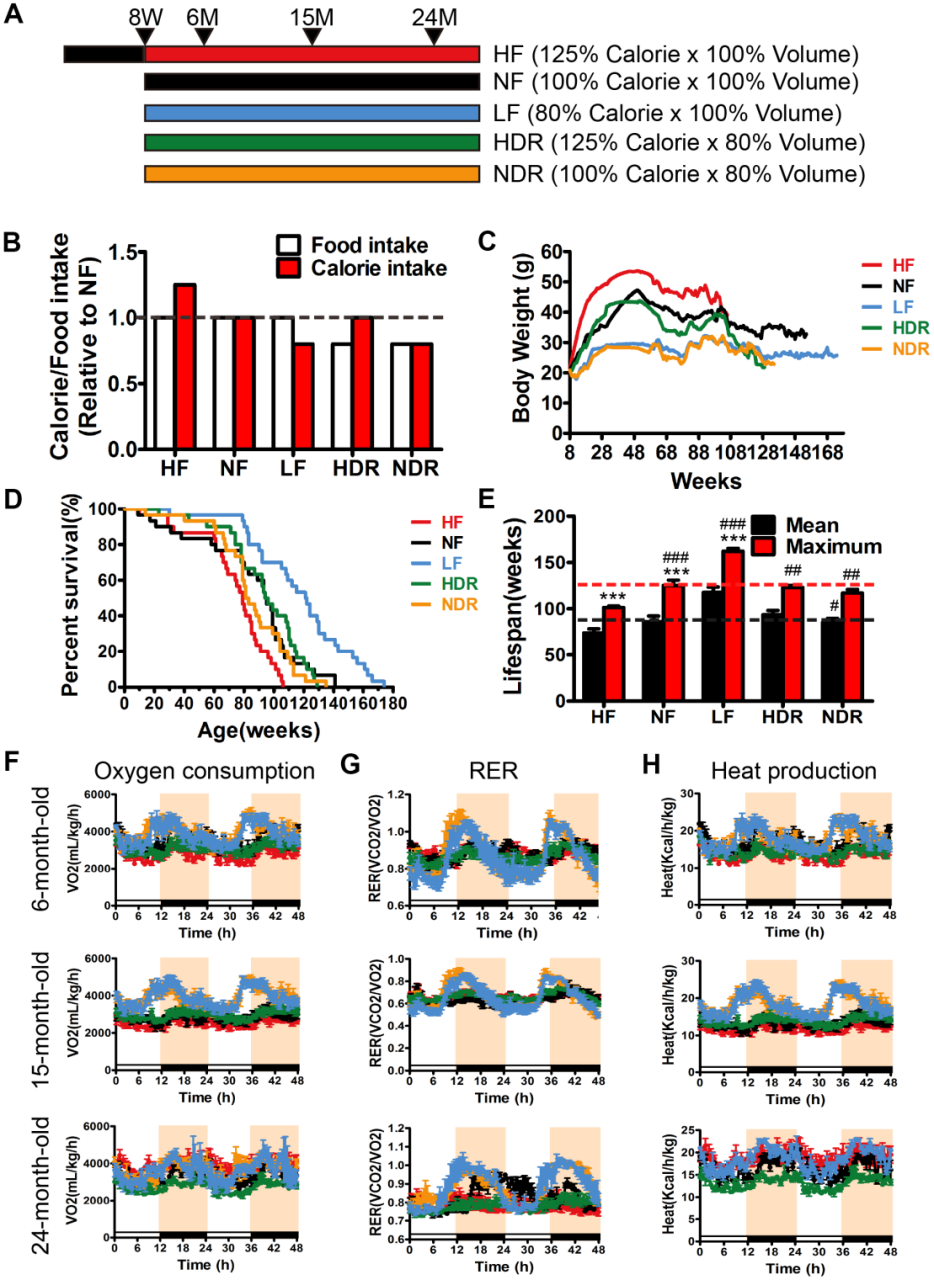
**Study design, lifespan and metabolic phenotypes of mice under different dietary regimens.** (**A**) The study design. NF mice had ad libitum access to normal chow. HF mice had ad libitum access to a high-calorie diet. (**B**) Food intake and calorie intake were calculated based on the daily food consumption of mice during the intervention (n=30 per group). (**C**) A line plot showing the average weekly weight in grams (mean ± SEM) of mice under different conditions (n=30 per group). HF mice gained weight, whereas HDR mice, despite being on a high-calorie diet, were indistinguishable from NF controls. (*P<0.05, compared to group NF). (**D**) Kaplan-Meier survival curves (n=30) showing significant difference in the lifespan between interventions according to the log-rank test (P<0.001). (**E**) Mean and maximum lifespans of mice in the five intervention groups as calculated from the Kaplan-Meier survival curves. The maximum lifespan was calculated as the average of the oldest 20% of mice in each group. (**F**) Oxygen consumption, (**G**) respiratory exchange ratio (CO_2_ exhaled/O_2_ inhaled), and (**H**) heat production at different ages (n=5 per group). ^#^*P*<0.05, ^##^*P*<0.01, ^###^*P*<0.001 vs the HF group; ^*^P<0.05, ^**^P<0.01, ^***^P<0.001 vs the NF group according to ANNOVAS.

To test whether food quantity-only or calorie-only restriction extends the lifespan, we assigned 8-week-old male C57BL/6N mice to five diets (n=30 per group). Detailed study design (diet composition, mouse numbers, and ages, and duration of the study) is shown in Supplemental Experimental Procedures, Figure 1A, and in Supplementary Tables 1, 2. Physiological studies have disclosed the expected beneficial effects of quantity restriction and calorie restriction on the animals’ body weight throughout their lifetime. Notably, restricting only calorie intake markedly reduced the weight gain with aging, whereas simple restriction of food quantity consumed had no effect on body weight (Figure 1B, 1C). The order of body weights among the groups was as follows: HF > NF ≈ HDR > LF ≈ NDR (Figure 1C). The Kaplan-Meier survival curves (Figure 1D) showed that the ranking of the mean and maximum lifespans (weeks) under the five dietary regimens as below: HF (mean=74.2, 101.5±1.5 maximum) < NF (mean=85.9, 125.3±1.9 maximum) < NDR (mean=85.3, 117±1.9 maximum) < HDR (mean=93.6, 123.2±2.1 maximum) < LF (mean=117.7, 162.2±1.5 maximum, Figure 1E). Thus, calorie restriction without restricting food quantity intake (LF group) is the most significant intervention for aging prevention and lifespan extension in this model. Our results also indicated that body weight and survival outcomes respond differently to calorie restriction.

To investigate the influence of energy balance on longevity, we calculated the daily energy intake of 15-month-old mice from the five groups and yielded the following results: HF > NF = HDR > LF = NDR. Energy expenditure was also measured in 15-month-old mice using a comprehensive laboratory animal monitoring system. For the reason that the body sizes of mice varied individually, we compared the metabolic rates by normalizing the data with the body weight of each mouse. Calorie-restricted mice manifested circadian rhythms in the oxygen consumption, systemic respiratory exchange ratio, and heat production (Figure 1F, 1H). Food quantity restriction alone had no significant effect as compared with NF mice. Additionally, calorie restriction increased physical activity during the light cycle in the LF group and decreased body temperature in LF and NDR groups at the age of 15 months (Supplementary Figure 1).

### Calorie restriction alone attenuates fat accumulation and related inflammation throughout the body

**Figure 2:**
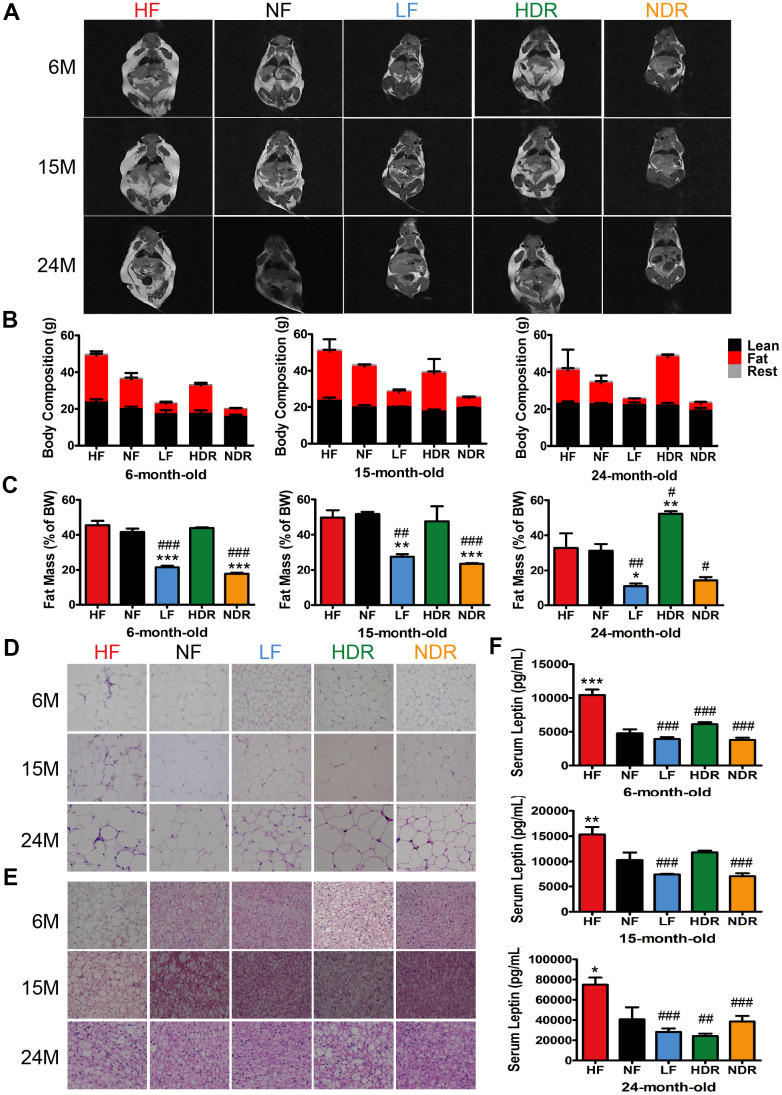
**Effects of calories and food quantity on the lifespan and energy state.** (**A**) *T_1_*-weighted MRI images of mice, where the highlighted part represents body fat. (**B**) Body composition of mice in each dietary group at different ages, and the corresponding (**C**) fat mass as a percentage of total body weight. Representative H&E-stained histological sections of (**D**) eWAT and (**E**) BAT under the different feeding conditions, as indicated. (**F**) Serum leptin concentration (n=6 per group). Data are presented as mean±SEM, ^#^*P*<0.05, ^##^*P*<0.01, ^###^*P*<0.001 vs the HF group; ^*^P<0.05, ^**^P<0.01, ^***^P<0.001 vs the NF group according to ANNOVAS.

Small animal magnetic resonance imaging (MRI) was used to determine the body composition of mice of all ages. Variations in fat mass explained the change in overall body weight, while lean mass was identical for all the experimental groups (Figure 2A–2C). In comparison with ad libitum-fed high-calorie or normal-calorie diets (HF or NF), calorie restriction reduced the levels of fat mass, whereas food intake seemed to have no effect on fat mass (Figure 2C and Supplementary Figure 2). These results indicated that calorie plays a decisive role in body fat reduction by calorie restriction. histological analyses of adipose tissue showed that calorie restriction resulted in a significant reduction in overall fat content. Hematoxylin and eosin (H&E) of epididymal white adipose tissue (eWAT) revealed smaller lipid drops in LF and NDR mice than in NF mice (Figure 2D). Further, large unilocular fat droplets significantly accumulated in brown adipose tissue (BAT) of HF mice, which were nearly absent in LF and NDR mice (Figure 2E). Besides, serum leptin levels were positively correlated with total body fat content. As predicted, serum leptin levels were lower in all calorie-restricted mice comparing to NF mice (Figure 2F).

In mice with diet-induced, fat accumulation in adipose tissue is associated with severer inflammation [26]. Consequently, H&E-stained eWAT sections displayed the typical coronal structure in HF mice but not in calorie-restricted mice (Figure 2D, arrowheads). Besides, mRNA levels of *TNFα* and *IL1β* in eWAT and BAT indicated reduced inflammation in the adipose tissue of calorie-restricted mice (Supplementary Figure 3A, 3B). Expressions of *Pgc1α* and *Ucp1* were upregulated in calorie-restricted mice (Supplementary Figure 3C, 3D). Our results showed that calories are a key determinant of obesity, and restriction of food quantity alone could hardly resist obesity.

### Effects of quantity or calorie restriction on motor coordination, memory, glucose tolerance, and insulin resistance

**Figure 3:**
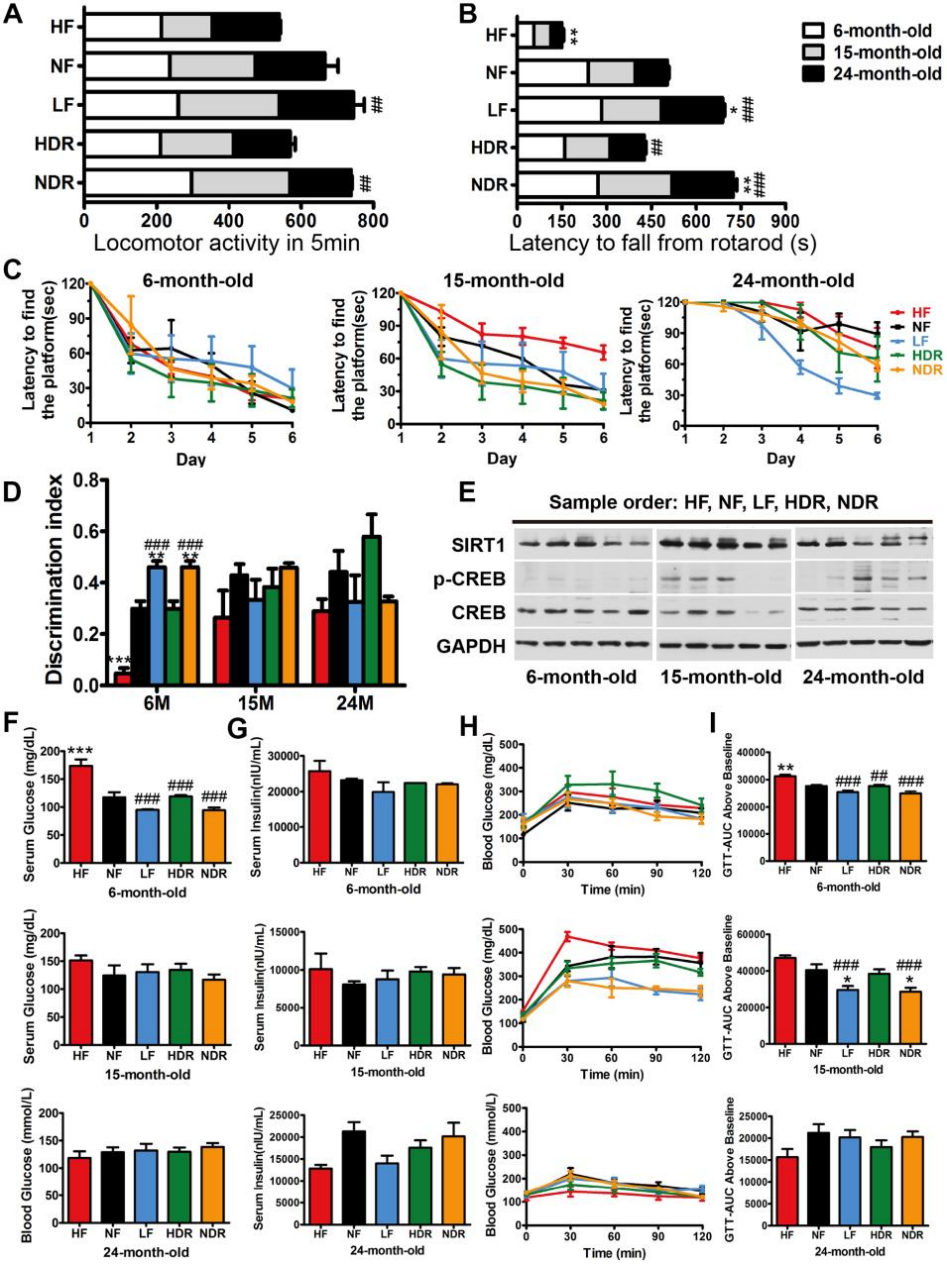
**Aging-sensitive markers, physical activity, and glucose tolerance of mice.** (**A**) Locomotor activity of mice at 6, 15, and 24 months of age (n=10 per group). (**B**) Latency to fall from a rotarod indicated the motor coordination of mice. (**C**) The results of the Morris water maze test for the mice under different dietary regimens. (**D**) The recognition index in the novel-object recognition test. (**E**) Representative immunoblots and densitometric quantification of the immunoblots for phosphor-Ser133-CREB (pCREB) in the mouse liver. (**F**) Serum glucose concentration in the different experimental groups and the corresponding (**G**) serum insulin concentrations (n=5 per group). (**H**) GTTs in the different experimental groups and the corresponding (**I**) areas under the curve (AUC). Five mice per group were analyzed. Data are presented as mean±SEM, ^#^*P*<0.05, ^##^*P*<0.01, ^###^*P*<0.001 vs the HF group; ^*^P<0.05, ^**^P<0.01, ^***^P<0.001 vs the NF group according to ANNOVAS.

Aging is related to an attenuation of locomotor and cognitive function (Figure 3A–3D) [27]. Interventions LF and NDR improved locomotor activity and performance on a rotarod test, which did not correlate with body weight (Figure 3A, 3B). It is presently unclear how far the healthspan effects of calorie restriction (e.g., enhanced performance on memory tasks) might extend within the lifespan. Thus, it is critical to determine whether the extended lifespan is associated with increases or deficits of cognitive capacity, therefore, we carried out a Morris water maze test at 6, 15, and 24 months of age (Figure 3C, 3D). There was an age-dependent impairment in learning and memory that was attenuated by calorie restriction, as measured by the latency to reach a platform. Short-term memory and context-dependent memory were investigated in the novel-object recognition tasks [28, 29]. LF and NDR mice showed higher recognition indexes compared to controls (Figure 3D). The mice spent more time exploring new objects, whereas no change in the total exploration time, indicating improvements in short-term cognitive performance, but not in general activity. CREB is an effector of neurotrophic factors that accounting for several neurodegenerative diseases associated with aging and brain responses to calorie restriction. The CREB-SIRT1 axis is an important component of the nutrient-sensitive molecular network associating calorie intake and energy metabolism with brain health [30], so we assessed the total amount and phosphorylation of the CREB and the expression of SIRT1, which is known to be a direct transcriptional target of CREB [31]. The expression of CREB was not modified by our dietary regimens; however, the phosphorylation of CREB on Serine 133 increased in the hippocampi of the LF group (Figure 3E), which suggested that the memory improvement was related to the activation of the CREB-SIRT1 axis.

Because calorie restriction attenuated fat accumulation and adipose inflammation, we next investigated whether it can protect from obesity-related insulin resistance and type II diabetes. We found that the levels of fasting glucose were higher in the HF groups compared with controls, but neither calorie restriction nor quantity restriction affected fasting glucose levels (Figure 3F). Compared with the regimen NF, fasting serum insulin contents were slightly lower by regimens LF and NDR (Figure 3G). The glucose tolerance test (GTT) showed improved glucose-tolerant of all the mice subjected to calorie or quantity restriction than that of NF group (Figure 3H, 3I). Besides, solely calorie restriction alone improved glucose tolerance, and this effect was similar to that of the simultaneous quantity and calorie restriction. Our results indicated that calories are the critical factor of all dietary restriction paradigms, whereas the effect of food quantity was not proved.

### Calorie restriction alone prevents immoderate body weight gain, hepatic steatosis, and liver disorders

**Figure 4:**
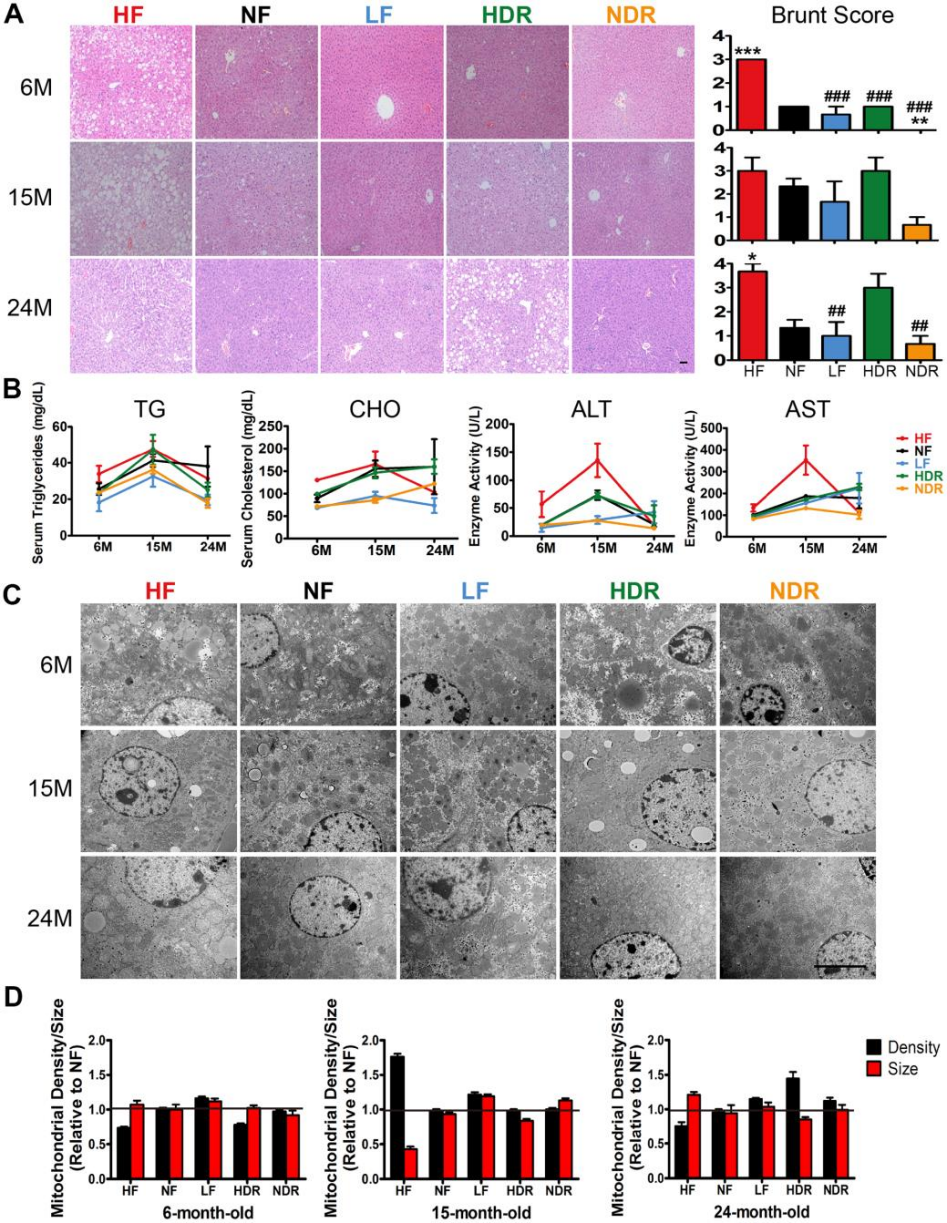
**Liver functions affected by the dietary interventions.** (**A**) Representative images of liver sections of mice of from each indicated group (n=5 per group) that were subjected to H&E staining. Original magnification: 20×. Steatohepatitis was scored by a histopathologist (who was blinded to the source condition of each sample) by a semiquantitative method derived from a published procedure [32]. (**B**) Liver function was assessed via the serum concentrations of triglycerides, cholesterol, ALT, and AST. (**C**) Transmission electron microscopy images of the liver in different experimental groups. Representative images at a magnification of 10,000× are shown. (**D**) Mitochondrial density and size. Mitochondrial densities were determined by normalizing the number of counted mitochondria to the area of each randomly selected cell (n=30 cells per group). Quantification of mitochondrial size was performed on five mice from each group. Data are shown as mean ± SEM, ^#^*P*<0.05, ^##^*P*<0.01, ^###^*P*<0.001 vs the HF group; ^*^P<0.05, ^**^P<0.01, ^***^P<0.001 vs the NF group according to ANNOVAS.

Compared to the normal diet ad libitum regimen (NF), calorie restriction alone (LF group) resulted in a lower weight; similar results were obtained in the NDR group. Nonetheless, the effect of quantity restriction alone was not remarkable (Figure 1C). The excessive increase in adiposity induced hepatic steatosis. In order to identify the pathological state and hepatic steatosis of mice in the five groups, we chose the Brunt Scoring System under blinded conditions. We found that hepatic steatosis was obviously reduced in LF mice in contrast with NF and HF mice (Figures 4A, Supplementary Figure 4A, 4B). Moreover, hepatic steatosis and injury markers (including total cholesterol, triglycerides, aspartate aminotransferase [AST], and alanine aminotransferase [ALT]) were obviously improved in LF mice (Figure 4B). Additionally, quantitative analysis of electron microscopy images showed no significant decrease in intracellular fat deposits, mitochondrial density, or endoplasmic reticulum in the livers of LF mice compared to the NF mice (Figure 4C, 4D).

### Kidney health in mice under different dietary regimens

To study the effects of different dietary regimens on aging, renal tissues were examined by routine H&E staining. Significantly higher numbers of dilated glomeruli and greater swelling of the renal tubule epithelium were noted in the HF and NF mice during aging; these parameters are well-known hallmarks of aging (Supplementary Figure 5A). Calorie restriction alone and, separately, quantity restriction alone alleviated the kidney fat lesions. Serum creatinine and urea nitrogen did not manifest significant differences at 6 and 15 months of age. However, compared with the NF mice at 24 months of age, serum creatinine levels in the LF, HDR, and NDR mice all significantly decreased, indicating protection from renal injury (Supplementary Figure 5B, 5C). Kidneys are the most important excretion organs of the body, and the main functions of kidneys are to filter the liquid forming urine, to excrete metabolic waste, and to regulate the balance of electrolytes and acid-base balance in the body. Our results revealed protective effects of the restrictions on calories and on food quantity against renal injury and inflammation.

### Midlife hepatic gene expression profiles under different dietary interventions

**Figure 5:**
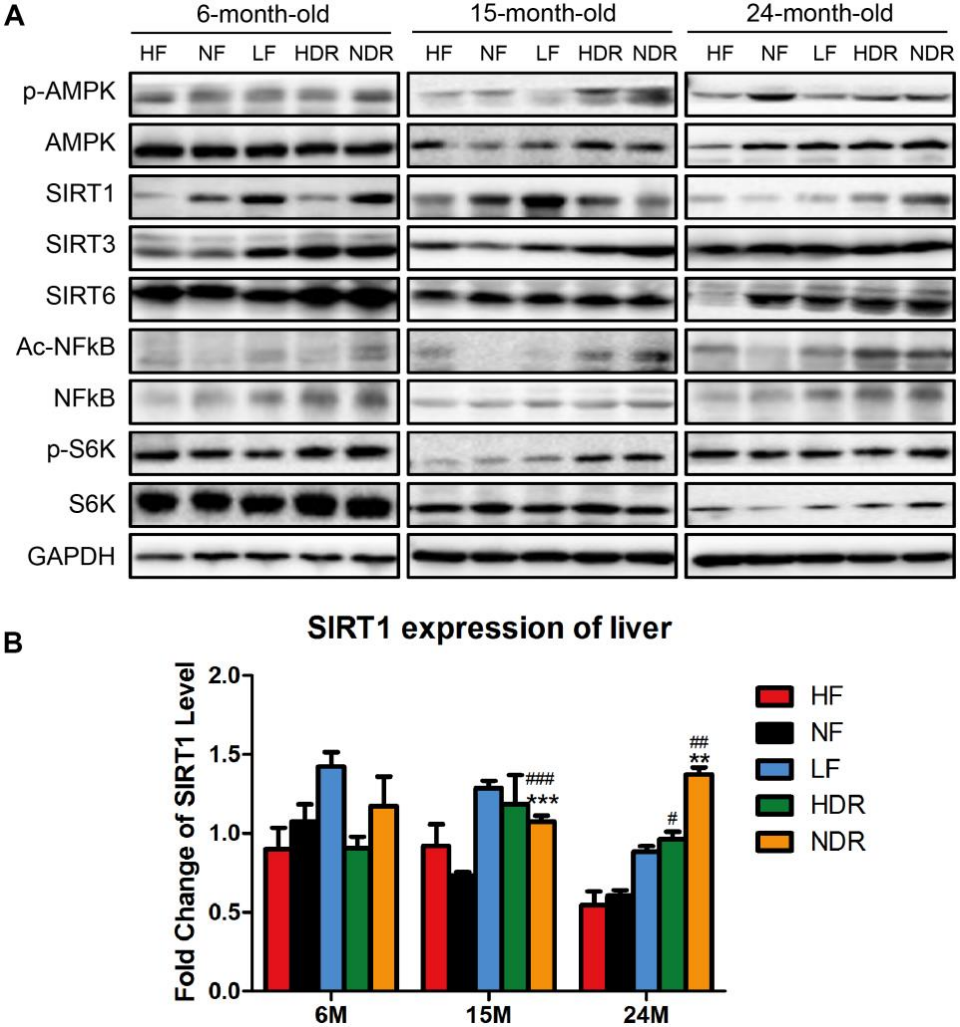
**Quantitative analysis of the expression of proteins related to classic pathways of calorie restriction.** (**A**) Western blotting analysis of SIRT1, SIRT3, SIRT6, phospho-AMPK, Ac-NFĸB in the liver of mice from different experimental groups (n=3 per group). (**B**) Immunohistochemical analysis of SIRT1 and Ac-NFĸB expression in the liver. ^#^*P*<0.05, ^##^*P*<0.01, ^###^*P*<0.001 vs the HF group; ^*^P<0.05, ^**^P<0.01, ^***^P<0.001 vs the NF group according to ANNOVAS.

Because calorie restriction is known to activate AMPK and SIRT1 or inhibit the mTOR pathway, we quantified the relevant signaling (including the ratio of phosphorylated S6K compared to total S6K) in the livers of the four groups of mice and found that the fold change of SIRT1 was most strongly related to the maximum lifespan (Figure 5A, 5B), suggesting that SIRT1 is the key protein for the lifespan extension by calorie restriction.

**Figure 6:**
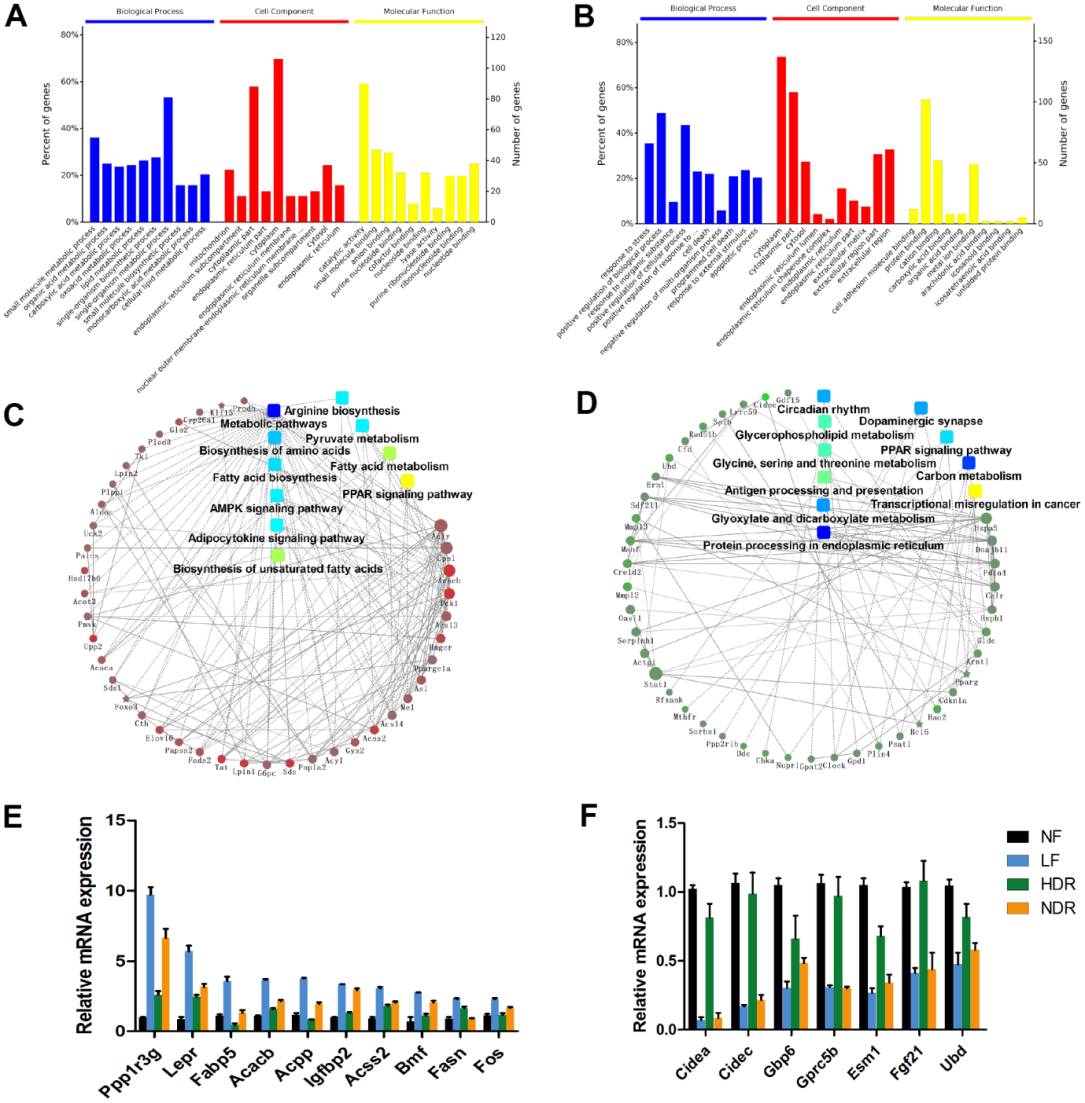
**Midlife liver gene expression correlations with lifespan are predictive of aging regulators.** (**A**) GO analysis of target genes with expression levels that are most positively correlated with the lifespan. (**B**) GO analysis of target genes with expression levels that are most negatively correlated with the lifespan. (**C**) Gene network modules depicting the pathways and genes that are most positively correlated with the lifespan. (**D**) Gene network modules showing the pathways and genes that are most negatively correlated with the lifespan. (**E**) mRNA expression of genes that are significantly positively correlated with the lifespan. (**F**) mRNA expression of genes that are significantly negatively correlated with the lifespan.

Nevertheless, the lifespan extension by calorie restriction is related to complex pathways and processes. To investigate the molecular mechanisms underlying physiological and lifespan distinctions in five dietary interventions, we determined the hepatic transcriptome of 3 mice in each group at 62 weeks of age by means of whole-genome microarray analysis. Among the five regimens, calorie restriction alone (LF) was the most effective intervention for the extension of healthspan and maximum lifespan; therefore, we ranked the mRNA expression changes according to the LF/NF ratio. Via this selection criterion, we found the genes whose expression showed the highest positive and negative correlations with the lifespan. Gene Ontology (GO) and KEGG pathway analyses suggested that these genes are related to various biological processes, including upregulated biosynthesis and metabolism of the fatty acid, biosynthesis of unsaturated fatty acids and amino acids including arginine (Figure 6A, 6C). Several other enriched pathways included carbon metabolism, glyoxylate and dicarboxylate metabolism, glycerophospholipid metabolism, and glycine, serine, and threonine metabolism (Figure 6B, 6D). In addition, a protein-protein interaction network was conducted to recognize core regulatory genes by means of microarray expression profiles. Reverse-transcription quantitative PCR confirmed that the expression of genes involved in fatty acid biosynthesis and metabolism, such as *Ppp1r3g*, *Fabp5*, *Fasn*, and *Fos*, significantly positively correlated with the lifespan (Figure 6E). The expression of genes related to carbon metabolism, including *Gbp5*, significantly inversely correlated with the lifespan (Figure 6F).

## DISCUSSION

In this research, we clarified that dietary interventions resulted in accordant alterations in aging-associated physiological and physical characteristics. Food quantity restriction alone did not yield a significant improvement in aging-related parameters, including body weight, inflammation, hepatic steatosis, and behavior test results. However, food quantity restriction seemed to attenuate the lifespan extension by calorie restriction. Simultaneously restriction of the food quantity and calories (NDR group) extended the healthspan, without a significant influence on the lifespan. That is to say, in mice, the health and survival outcomes in response to calorie restriction are different to a certain extent. It is presently unclear how far the healthspan effects of calorie restriction (e.g., enhanced performance on memory tasks) might extend within the lifespan. Mice under these dietary regimens may consume more energy to sustain their healthspan, thus shortening their lifespan.

We tried to keep the total calories the same when adjusting the food quantity. Inevitably, we had to use diets with different compositions, such as corn sugar, soy oil, or lard oil, which leads to two variables in the diets including composition and quantity, when the total calories are the same. If the quantity is reduced, the fat has to be increased to keep the same calories and vice versa. However, it was reported that daily calorie intake per se was identified as a determinant factor of the lifespan, and the source of calories (carbohydrates, fat, or protein) was considered irrelevant [11]. So it is reasonable that we didn’t take the influence of the source of calories into consideration in our experiments. Even though we considered the variable “composition”, the calories intake of the LF and NDR groups are the same, and the variables that account for the lifespan difference between these two groups are food quantity and diet composition. The proportion of fat (soy oil) in the diet of NDR group is higher, and as we know, soy oil contains large quantities of saturated fatty acids which is good for health. The shorter lifespan extension of NDR compared to LF still proved that quantity restriction probably attenuated lifespan extension of calorie restriction.

The effects of calorie restriction are age-dependent. The effects of different dietary regimens on aging-related characteristics varied widely among different ages throughout the lifespan. For example, the data on learning abilities and memory of mice under different regimens are quite contradictory among different age groups, that is to say, calorie restriction did not improve learning and memory in 6- and 15-month-old mice but it significantly improved that of 24-month-old mice. In the novel object recognition task, only young-aged calorie-restricted mice significantly spent more time exploring new objects, without significant differences (from the control) at the middle age and old age. This difference may perhaps be due to the difference in sensitivity between the Morris water maze and novel object recognition tests. Future studies would be warranted to make these distinctions.

Though calorie restriction is reported to extend longevity and prevent a number of chronic diseases, the underlying metabolic mechanisms are poorly understood. Several hypotheses have been advanced to explain biologically how calorie restriction prolongs the lifespan and reduces aging-related problems. The most frequently reported effects include growth retardation, loss of body fat, attenuated hormonal and immunological changes, enhanced damage repairability, activation of autophagy and apoptosis, altered gene expression, activation of sirtuins, altered IGF/insulin/TOR signaling, decreased body temperature, and attenuation of oxidative stress. However, the core issue, specifically, how calorie restriction counters the effects, remains doubtful. We detected changes in the protein expression of these classic pathways involved in the effects of calorie restriction and found that SIRT1 is one of the critical proteins related to lifespan.

The increased healthspan of mice with calorie restriction alone likely reflects better metabolic responses mobilizing energy stores [33]. The most important changes related to the lifespan were the following: calorie restriction improved fatty acid biosynthesis and metabolism. Considering the changes in respiratory exchange ratio, calorie-restricted mice oxidized a greater quantity of fatty acids per day (than food quantity-restricted mice) but showed stable weight. We concluded that fatty acid biosynthesis accounts for a high proportion of whole-body fatty acid metabolism during excessive dietary fat intake. Calorie restriction alone also improved the biosynthesis of amino acids, and arginine is the precursor of the three pathways, and its products are related to tissue damage and repair. In addition, calorie restriction alone inhibited carbon metabolism as well as glyoxylate and dicarboxylate metabolism. The shift from carbon metabolism to fatty acid biosynthesis and metabolism in the body may represent the key changes related to the extension of the healthspan and lifespan.

Ultimately, our results highlight a great potential for calorie restriction to counteract aging and aging-associated metabolic disorders. Low-calorie food at normal food intake is healthier and more effective at preventing aging and aging-related diseases than calorie restriction at food intake restriction. That is, eating low-calorie food is better than eating less. This work has significant implications for the application of calorie restriction to humans.

## MATERIALS AND METHODS

### Animals and diets

All the animal experiments were conducted according to the guidelines of the Institutional Animal Care and Use Committee of Tsinghua University. 6-week-old male C57BL/6N mice were obtained from Vital River Laboratories. The mice were fed in a 12:12 light/dark cycle with AIN-93G food ad libitum for 2 weeks. After that, the mice were randomly divided into different groups with distinct feeding regimens, and the detailed description was shown in Supplementary Figure 1, and Supplementary Table 2 of Supplemental Experimental Procedures. The diets used in this research were as follows: a normal-calorie diet (AIN-93G diet), a 125%-calorie diet, and an 80%-calorie diet. All the mice were individually housed. We monitored the food intake and body weight weekly throughout the experiment. The daily consumption of food in NF group over 1 week was recorded and averaged to quantify the food quantity of the LF, HDR, and NDR groups for the subsequent weeks. Food for these groups was given at 2 pm daily that ensures isocaloric food consumption between LF and NDR groups, or between NF and HDR groups.

### Determination of the lifespan in mice

Survival was investigated among the mice (n=30 per group), that were under careful observation daily regarding deaths. Survival curves were plotted according to the Kaplan-Meier method, and a log-rank test was used to evaluate the differences in lifespan among different dietary interventions. Maximum lifespan was counted as the average age of the oldest 20% of mice within each group.

### Metabolic cages

According to the manufactures’ guides, the whole-body metabolism was measured with the indirect calorimetry in a CLAMS system (Columbus Instruments) for 2 days following 4 days of adaptation. Feeding and light conditions remained consistent with in-home cages.

### Behavioral tests

Locomotor activity and the latency time to fall from an accelerating rotarod were measured under each dietary intervention to evaluate motor coordination (Chinese Academy of Medical Sciences). The Morris water maze test and the latency to fall from a string test were conducted using the facilities at the Center for Life Sciences of Tsinghua University [34].

### Glucose tolerance testing

Mice fasted for 16 hr with access to water ad libitum. Fasting glucose levels were measured by the Glucometer (One Touch Ultra Easy) via tail bleeds. Next, the mice were injected with glucose intraperitoneally at 2 g/kg of body weight, and blood glucose was measured every 30 min for 2 hr [18].

### Body composition

Body fat and lean mass of live mice were measured in a mouse MRI instrument (Echo Medical Systems) following the manufacturer’s instructions.

### Histochemical section observation

Sections of the liver, kidney, and adipose tissue were stained by hematoxylin and eosin; liver sections were also stained by Sirius red. Then we performed the observation using a light microscope (Leica).

### Quantitative RT-qPCR assay

RNA was extracted from eWAT, BAT, or liver tissues using Trizol LS Reagent (Invitrogen) and purified with RNAEasy mini kit (Qiagen). Primers obtained from Invitrogen were used for gene quantification with the CFX96TM Real-Time System, which was conducted in triplicate.

### Western blotting

Samples for Western blotting were extracted using RIPA lysis buffer supplemented with a cocktail of protease and phosphatase inhibitors. The same quantity of protein was loaded on 10% SDS-PAGE and transferred to PVDF membranes. The following antibodies were used: SIRT1, SIRT3, p-CREB, CREB, p-AMPK, AMPK, Ac-NFkB, NFkB, p-S6K, S6K, GADPH (Cell Signaling Technology), and SIRT6 (Abgent).

### Microarray analysis

The hepatic transcriptional levels of genes in three mice from each group were analyzed by means of the Affymetrix Mouse Genome 432 2.0 Array at 62 weeks of age. We log_2_-transformed and normalized the raw data utilizing the affy package in Bioconductor. Expressed genes variations among different dietary interventions were determined in the RankProd software [35] with the proportion of false positives (pfp) < 0.1. Among the five regimens, calorie restriction alone (LF) was the most effective intervention for the extension of healthspan and maximum lifespan; therefore we ranked the mRNA expression changes according to the LF/NF ratio. Via this selection criterion, we found the genes whose expression displayed the highest positive and negative correlations with the lifespan. GO and KEGG pathway analyses revealed the critical pathways correlated with lifespan. KEGG pathways were downloaded from the KEGG database on May 10, 2018. GSEA system was used for significance analysis. In addition, a PPI network was conducted to identify hub regulatory genes by means of the microarray expression profiles. RT-qPCR confirmed the expression of genes positively or inversely correlated with the lifespan.

### Data availability statement

All data are available in the manuscript or the supplementary materials. Correspondence and requests for materials should be addressed to corresponding author Z.W.

### Ethics approval statement

All animal experimental protocols were approved by Institutional Animal Care and Use Committee (IACUC) of Tsinghua University.

## Supplementary Material

Supplementary Figures

Supplementary Tables
