# Supplementary material for: Calorie intake rather than food quantity consumed is the key factor for the anti-aging effect of calorie restriction
Source: Aging (Albany NY). 2021 Sep 7;13(17):21526–46. doi: 10.18632/aging.203493 (PMC8457579; doi:10.18632/aging.203493)
Supplement: Supplementary Figures [file aging-13-203493-s001.pdf]

SUPPLEMENTARY FIGURES

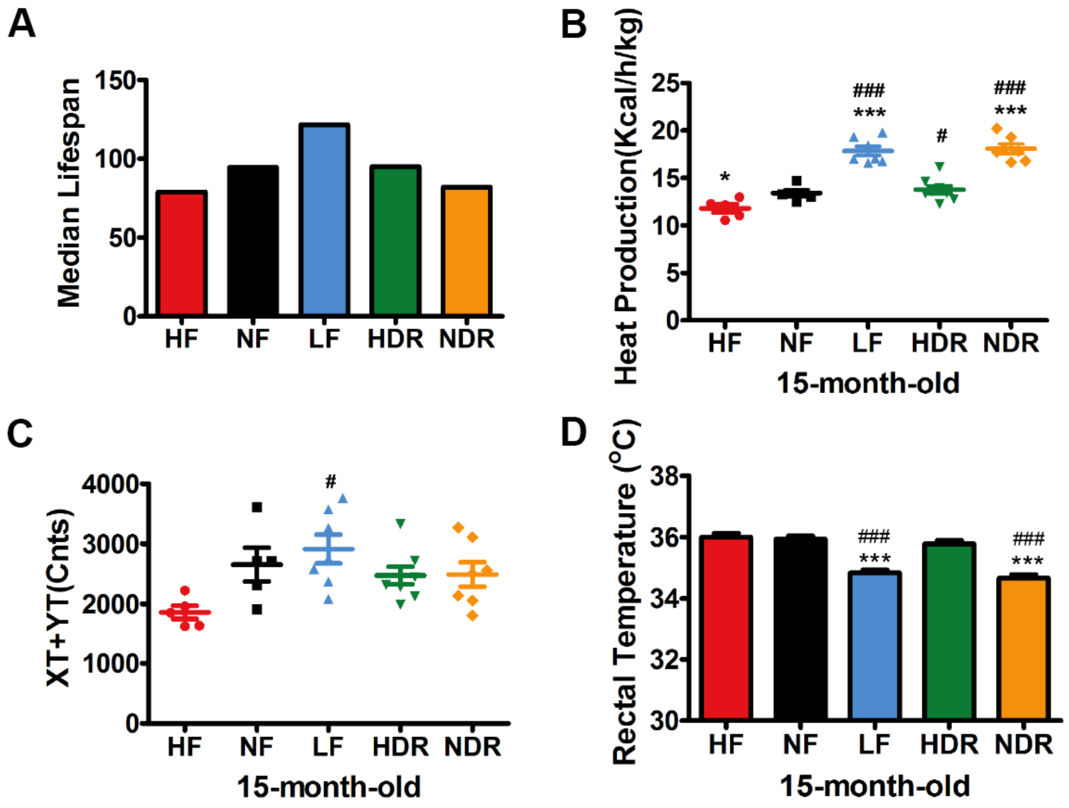

Supplementary Figure 1. Median lifespan, midlife physical activity and body temperature of mice. (**A**) Median lifespan of mice under five different dietary regimens. (**B**) Heat production of midlife mice under different dietary regimens. (**C**) Daily activity of midlife mice under different dietary regimens. (**D**) Rectal temperature of midlife mice under different dietary regimens. $^{\#}P<0.05$, $^{\#\#}P<0.01$, $^{\#\#\#}P<0.001$ vs the HF group; $^{*}P<0.05$, $^{**}P<0.01$, $^{***}P<0.001$ vs the NF group.

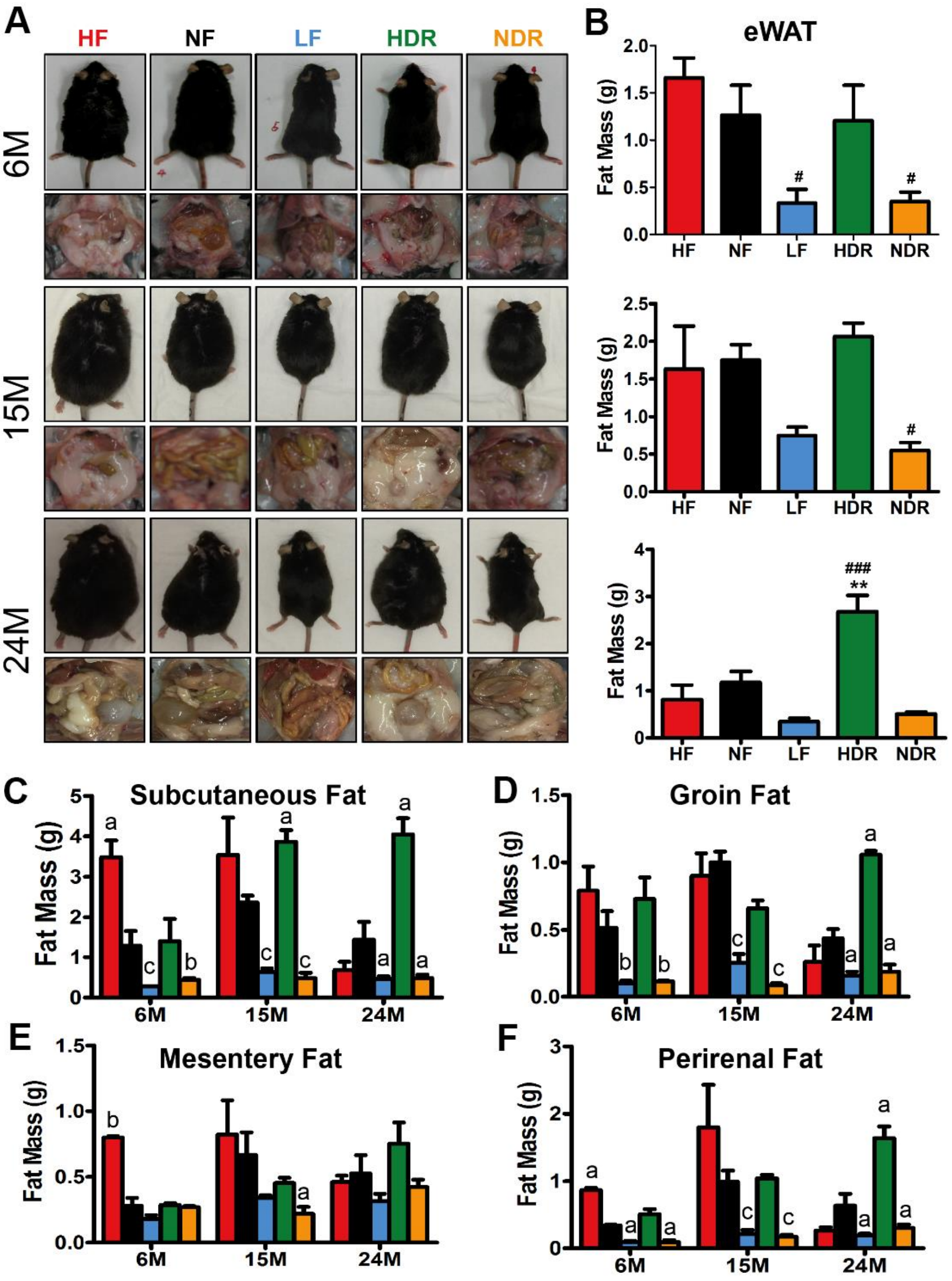

Supplementary Figure 2. Body fat distribution under different dietary regimens. (**A**) Pictures of randomly selected whole body (upper) and body cavity (lower) of mice under different dietary regimens. (**B**) Fat mass of eWAT at different ages. (**C**) Subcutaneous fat, (**D**) Groin fat, (**E**) mesentery fat and (**F**) perirenal fat mass under different dietary regimens. $^{\#}P$<0.05, $^{\#\#}P$<0.01, $^{\#\#\#}P$<0.001 vs the HF group; $^{*}P$<0.05, $^{**}P$<0.01, $^{***}P$<0.001 vs the NF group.

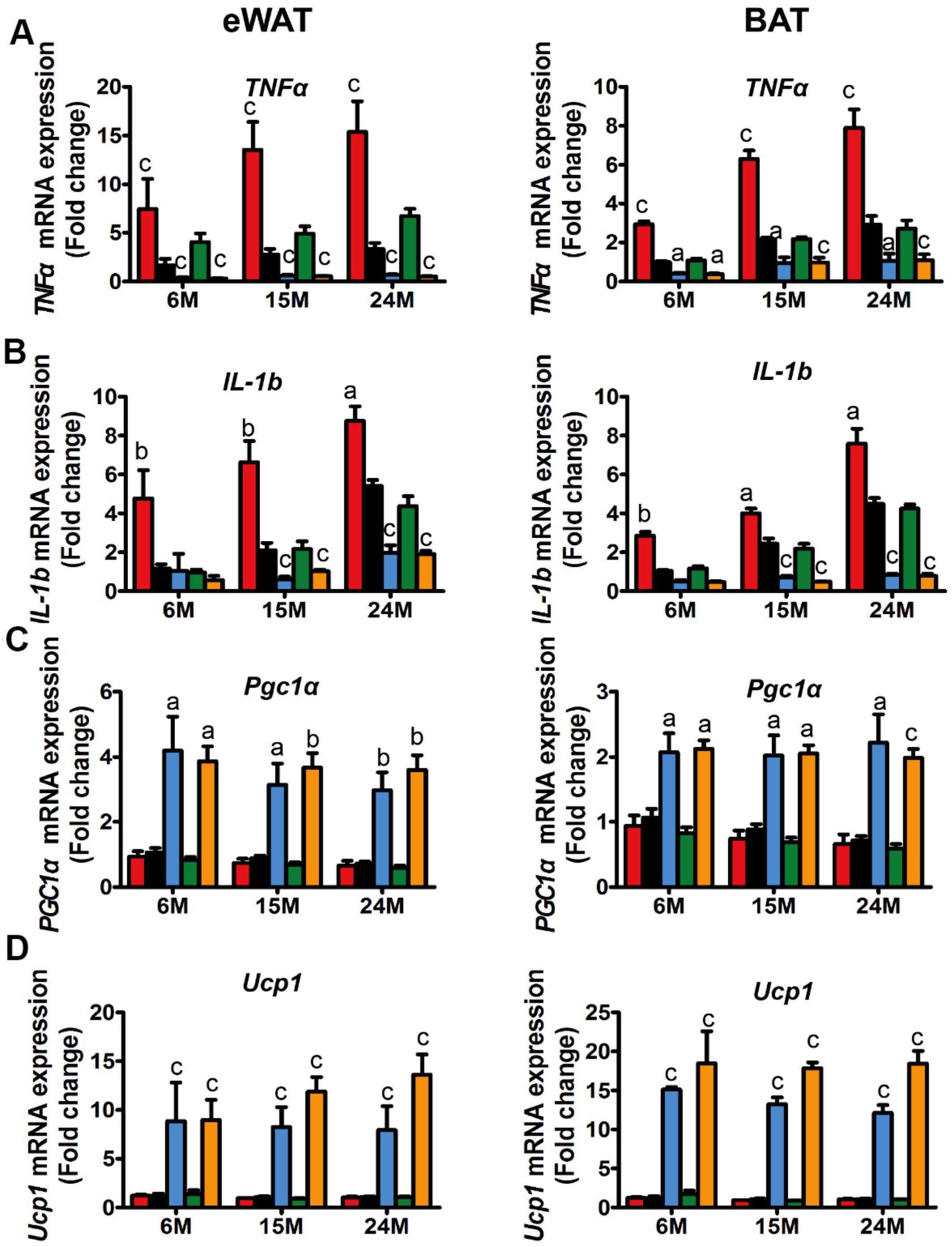

Supplementary Figure 3. mRNA quantification of selected pro-inflammatory cytokines and chemokines. (**A**) *TNFα*, (**B**) *IL-1b*, (**C**) *Pgc1α* and (**D**) *Ucp1* expression in eWAT and BAT under different dietary regimens. a, $P<0.05$; b, $P<0.01$; c, $P<0.001$ vs the corresponding NF groups.

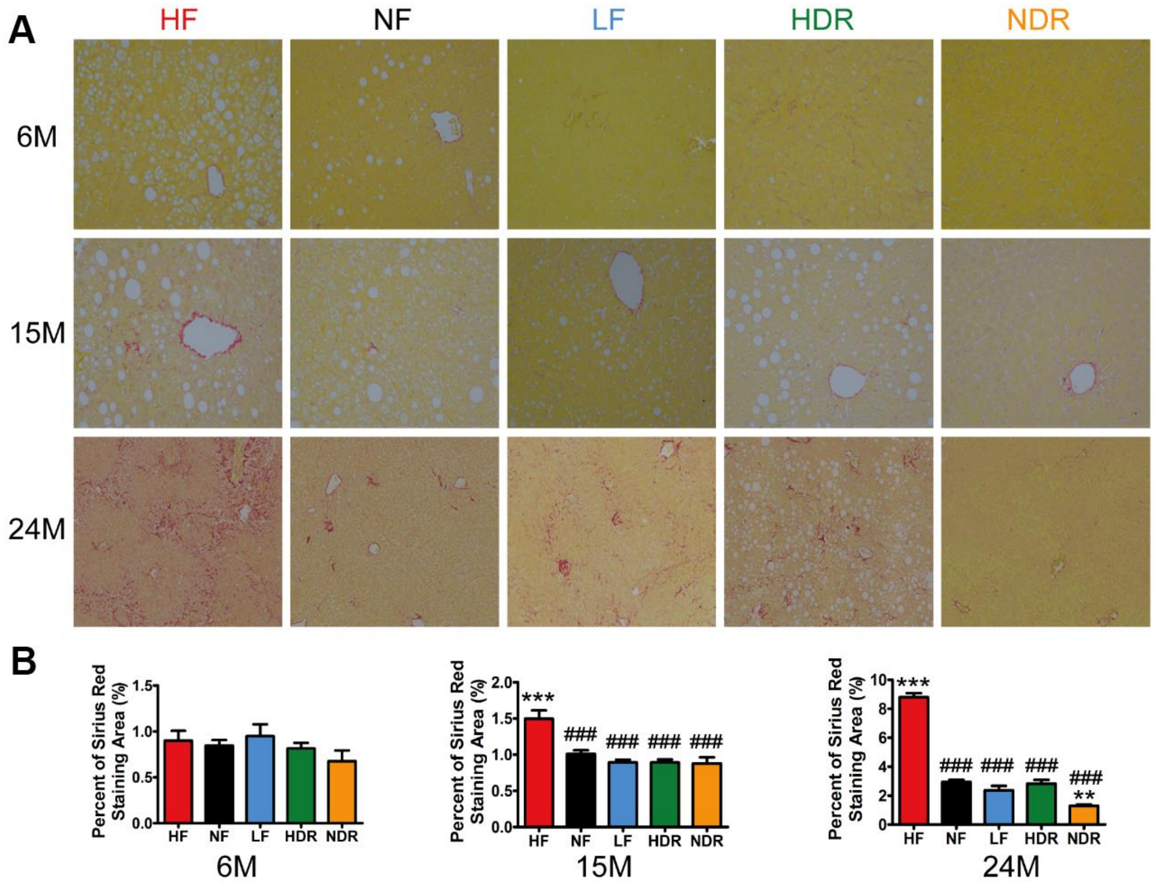

Supplementary Figure 4. Sirius red staining of liver in different dietary regimens. (**A**) Sirius red staining. (**B**) Percent of Sirius red staining area. $^{\#}P<0.05$, $^{\#\#}P<0.01$, $^{\#\#\#}P<0.001$ vs the HF group; $^{*}P<0.05$, $^{**}P<0.01$, $^{***}P<0.001$ vs the NF group.

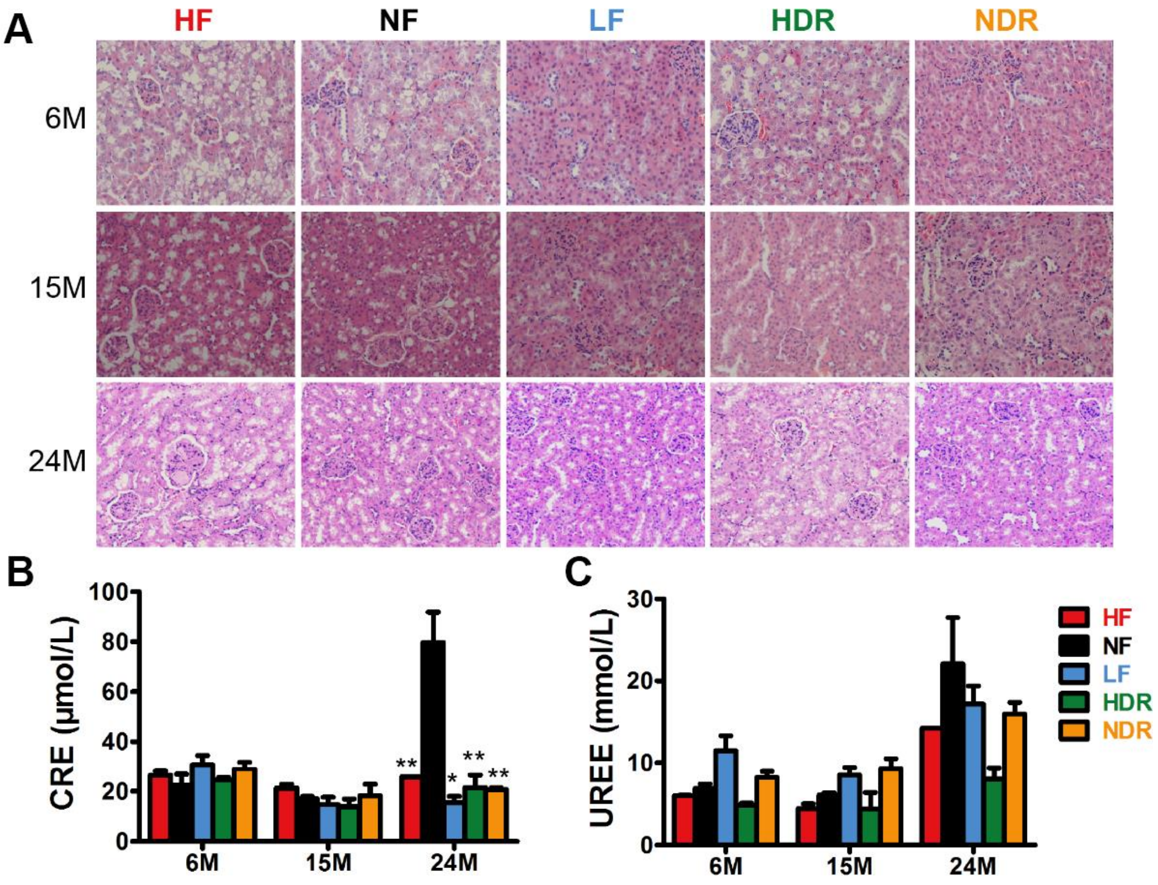

Supplementary Figure 5. Kidney functions affected by the dietary interventions. (**A**) Representative images of kidney sections of mice of each indicated group (n=5 per group) after H&E staining. Original magnification: 20×. (**B**) The extent of kidney injury was scored by a histopathologist (who was blinded to the source condition of each sample) by a semiquantitative method. (**C**) Kidney function was assessed by means of serum concentrations of creatinine and urea nitrogen (n=6 per group). Data are shown as mean ± SEM, $^{\#}P<0.05$, $^{\#\#}P<0.01$, $^{\#\#\#}P<0.001$ vs the HF group; $^{*}P<0.05$, $^{**}P<0.01$, $^{***}P<0.001$ vs the NF group according to ANNOVAS.
