# Supplementary material for: Calorie intake rather than food quantity consumed is the key factor for the anti-aging effect of calorie restriction
Source: Aging (Albany NY). 2021 Sep 7;13(17):21526–46. doi: 10.18632/aging.203493 (PMC8457579; doi:10.18632/aging.203493)
Supplement: Supplementary Tables [file aging-13-203493-s002.pdf]

SUPPLEMENTARY TABLES

Supplementary Table 1. Formula of the different diets used in the study.

A. AIN-93G diet (100%).

Composition	Weight(g)	Energy(kcal/kg)
Corn Sugar	397.486	1589.944
Casein Lactic	200	800
Granular Sugar	100	400
Dextrin	132	528
L-Cystine	3	12
Solka Floc-40	50	0
Soy Oil	70	630
Antioxidants	0.014	0
AIN-93 Mineral Mix	35	0
AIN-93 Vitamin Mix	10	40
Choline Bitartrate	2.5	0
In total	1000	3999.944

B. High-calorie diet (125%).

Composition	Weight(g)	Energy(kcal/kg)
Corn Sugar	197.486	789.944
Casein Lactic	200	800
Granular Sugar	100	400
Dextrin	132	528
L-Cystine	3	12
Solka Floc-40	50	0
Soy Oil	70	630
Lard Oil	200	1800
Antioxidants	0.014	0
AIN-93 Mineral Mix	35	0
AIN-93 Vitamin Mix	10	40
Choline Bitartrate	2.5	0
In total	1000	4999.944

C. Low-calorie diet (80%).

Composition	Weight(g)	Energy(kcal/kg)
Corn Sugar	317.486	1269.944
Casein Lactic	200	800
Granular Sugar	100	400
Dextrin	132	528
L-Cystine	3	12
Solka Floc-40	180	0
Soy Oil	20	180
Antioxidants	0.014	0
AIN-93 Mineral Mix	35	0
AIN-93 Vitamin Mix	10	40
Choline Bitartrate	2.5	0
In total	1000	3229.944

Supplementary Table 2. Detailed characteristics of the animal cohorts used in this study.

Cohort	Calorie	Food quantity	Corn sugar	Soy oil	Lard oil
HF	125%	100%	197g	70g	200g
NF	100%	100%	397g	70g	0g
LF	80%	100%	317g	20g	0g
HDR	100%	80%	158g	56g	160g
NDR	80%	80%	317g	56g	0g
